# Potentiometric Urea Biosensor Based on an Immobilised Fullerene-Urease Bio-Conjugate

**DOI:** 10.3390/s131216851

**Published:** 2013-12-06

**Authors:** Kasra Saeedfar, Lee Yook Heng, Tan Ling Ling, Majid Rezayi

**Affiliations:** 1 Faculty of Science and Technology/SEADPRI, LESTARI, University Kebangsaan Malaysia, Selangor 43600, Malaysia; E-Mail: lingling@ukm.my; 2 Chemistry Department, Faculty of Science, K. N. Toosi University of Technology, Tehran 15418-49611, Iran; E-Mail: Kasra53@yahoo.com; 3 ACECR Mashhad Branch, Food Science and Technology Research Institute, Mashhad 91775-1376, Iran; E-Mail: chem_rezayi@yahoo.com

**Keywords:** fullerene, urease, urea biosensor, potentiometric

## Abstract

A novel method for the rapid modification of fullerene for subsequent enzyme attachment to create a potentiometric biosensor is presented. Urease was immobilized onto the modified fullerene nanomaterial. The modified fullerene-immobilized urease (C_60_-urease) bioconjugate has been confirmed to catalyze the hydrolysis of urea in solution. The biomaterial was then deposited on a screen-printed electrode containing a non-plasticized poly(*n*-butyl acrylate) (P*n*BA) membrane entrapped with a hydrogen ionophore. This pH-selective membrane is intended to function as a potentiometric urea biosensor with the deposition of C_60_-urease on the PnBA membrane. Various parameters for fullerene modification and urease immobilization were investigated. The optimal pH and concentration of the phosphate buffer for the urea biosensor were 7.0 and 0.5 mM, respectively. The linear response range of the biosensor was from 2.31 × 10^−3^ M to 8.28 × 10^−5^ M. The biosensor's sensitivity was 59.67 ± 0.91 mV/decade, which is close to the theoretical value. Common cations such as Na^+^, K^+^, Ca^2+^, Mg^2+^ and NH_4_^+^ showed no obvious interference with the urea biosensor's response. The use of a fullerene-urease bio-conjugate and an acrylic membrane with good adhesion prevented the leaching of urease enzyme and thus increased the stability of the urea biosensor for up to 140 days.

## 1. Introduction

In fullerene (C_60_), each carbon atom is bound to three other carbons and is *sp*^2^-hybridized. The C_60_ molecule has two types of bonds: the double bond of 6:6 ring bonds and the shorter bond in 6:5 bonds. C_60_ behaves like electron-deficient alkenes, and readily reacts with electron-rich species [1]. Their small size, inert behavior, and stable structure account for the low toxicity of fullerenes, even at relatively high concentrations. Their electrochemical characteristics combined with unique physiochemical properties enable the application of fullerenes in the design of novel biosensor systems. Given their possible protein and enzyme functionalization, as well as their signal mediation and light-induced switching, fullerenes can potentially provide new and powerful tools in the fabrication of electrochemical biosensors [2].

Urea is one of the metabolic products of protein metabolism. The accurate determination of urea is essential in glomerular filtration rate determination, renal function testing, and other biomedical applications. The normal level of urea in serum ranges from 15 mg/dL to 45 mg/dL (25 mM to 75 mM). The concentrations increase in the serum from 180 mg/dL to 480 mg/dL (300 mM to 800 mM) in patients suffering from renal insufficiency. The estimation of urea is likewise crucial in food science and environmental-monitoring. Urea has a strategic function in the marine nitrogen cycle as a source of excreted nitrogen by invertebrates and fish. Likewise, the bacterial decomposition of nitrogenous materials and terrestrial drainage are influenced by urea. Urea estimation is important during environmental monitoring. The annual worldwide production of urea exceeds 100 million metric tons, and the majority of which is used as fertilizer. Excessive nitrogen fertilizer application can lead to pest problems by increasing birth rate, longevity, and overall fitness of certain pests. Urea may be responsible for reduction in soil pH [3].

Various analytical methods for determining trace amounts of urea have been developed [3]. Optical [4], amperometric [5–7], thermal [8,9] conductometric [10], and potentiometric [11–15] methods are commonly used to measure urea in samples. Given the simple construction of potentiometric urea biosensors and the availability of the required instrumentation for their utilization, these biosensors are widely accepted [12–14,16,17].

The enzyme urease could be employed for urea determination, whereby the urease catalyzes the hydrolysis of urea to form alkaline reaction products. To construct a functional nanomaterial-based biosensor, the relationship between enzymes and nanomaterials such as fullerene and carbon nanotubes (CNTs) must be identified. The interaction between the enzyme and the biosensor could be a covalent or non-covalent bond. Several reports have identified the immobilization of biomolecules on CNTs via non-covalent interactions [18,19]. The improved stability, accessibility, and selectivity, as well as the reduced leaching, can be achieved through covalent bonding because the location of the biomolecule can be controlled [20].

Several types of immobilization methods for biological molecules are available. These methods include entrapment, encapsulation, covalent binding, cross-linking, and adsorption. Fullerene is an allotrope of carbon with 60 π electrons. Thus, fullerene resembles olefin molecules and can undergo nucleophilic attack by electron-releasing molecules such as amines. Fullerene has low solubility in aqueous solutions [21–24]. Fullerenes have been used in the fabrication of certain biosensors with enzymes such as lipase and urease. Lipase immobilized on fullerene was used to detect optical isomers of amino acid esters and urea at 10^−1^ to 10^−4^ M measured by the quartz crystal microbalance (QCM) method [21,23]. Urease possesses an amine (–NH_2_) group that can directly react with 30 π-bonds in fullerene. The transduction processes may be performed by optical, piezoelectric and amperometric methods. The absence of an organic solvent during the covalent immobilisation steps are some benefits in this research [21]. The difficult sample preparation for the QCM method and the expensive instruments for measuring urea have highlighted the benefits of the potentiometric method. The short lifespan, delayed response time, low sensitivity, and other limitations of the prepared membranes for the potentiometric method are some aspects that require further investigation.

Fullerene is expected to increase the sensitivity of the potentiometric method when it is combined with urease because of the high surface area-to-volume ratio of the nanomaterial for urease immobilization. In the present study, a new way to construct a urea biosensor has been developed. The fullerene nanomaterial was functionalized with carboxyl (–COOH) groups by sonication, heat, and ultraviolet (UV) radiation. Urease enzyme was immobilized onto –COOH-modified fullerenes (C_60_-COOH) in the presence of *N*, *N*′-dicyclohexylcarbodiimide (DCC) or *N*-(3-dimethylaminopropyl)-*N*′-ethylcarbodiimide hydrochloride (EDC). The immobilization process was characterized by Fourier-transform infrared spectroscopy (FTIR) and scanning electron microscopy (SEM). The fullerene-immobilized enzyme was then deposited onto a pH-selective screen-printed electrode (SPE) containing an acrylic membrane with good adhesion to fabricate a potentiometric urea biosensor for the quantitative determination of urea. The good adhesion of the fullerene-urease biomaterial on the acrylic membrane enables a long lifespan, high stability, and rapid response time of the urea biosensor compared with other membrane-based potentiometric urea biosensors.

## 2. Material and Methods

### 2.1. Materials and Instruments

Purchased fullerene from Aldrich (Saint Louis, MO, USA) was purified and functionalized with H_2_SO_4_ and HNO_3_. 2,2-Dimethoxy-2-phenylacetophenone (DMPP), *n*-butyl acrylate (*n*-BA), sodium tetrakis(4-flourophenyl)borate dehydrate (NaTFPB), EDC, and DCC were purchased from Fluka (Steinheim, Germany). 1,6 Hexanediol diacrylate was purchased from Aldrich (Saint Louis, MO, USA). Urease (U4002-100 KU, type IX) and bovine serum albumin (BSA) were obtained from Sigma–Aldrich. Phosphate-buffered solutions (PBS) were prepared by using K_2_HPO_4_ and KH_2_PO_4_ from Merck (Darmstadt, Germany). Hydrogen ionophore I (HI; tridodecylamine) was obtained from Fluka. Bactor agar was purchased from Ajax Chemicals (Scoresby, Australia) and tris(hydroxymethyl)aminomethane (Tris–HCl) was purchased from Duchefa Biochemie (RV Haarlem, Netherlands). All chemicals were of analytical grade and used without further purification. All solutions and standard buffer solutions were prepared with deionized water (18 μS/cm^2^).

Potentiometric measurement was performed using an Orion model 420 potentiometer. A hand-made Ag|AgCl electrode was used as the reference electrode. The procedures were characterized by field emission SEM (FESEM; Zeiss Supra 55 VP, Oberkochen, Germany), and variable pressure (VP) SEM–energy-dispersive x-ray (EDX) spectroscopy (VPSEM-EDX; Philips XL 30, Eindhoven, Netherlands). The characterization was performed using a Perkin Elmer Spectrum GX FTIR spectrometer (Wellesley, MA, USA). Samples were dried using a freeze-dryer (Christ, Osterode am Harz, Germany). Homogenous mixtures of fullerenes in strong acid solutions were prepared using an Elma S30H sonicator bath. The Ag|AgCl SPE was utilized as the working electrode.

### 2.2. Methods

#### 2.2.1. Surface Modification of Fullerene Nanomaterials

Surface modification of the fullerene nanomaterials was performed by adding 1 mL of concentrated H_2_SO_4_/HNO_3_/H_2_O (3:1:3) solution to 5.0 mg of unmodified fullerene, which was then oxidized for 90 min at 75 °C in a sonicator bath. Simultaneous sonication and UV radiation treatment of the mixture was performed for another 3 min. The influence of UV radiation on the surface-modified fullerene nanomaterial was examined by FTIR. The carboxylic acid-functionalized fullerene nanomaterial was then separated from the acid solution via centrifugation and neutralized with deionized water to a pH around 6. Thereafter, the modified fullerene nanomaterial was frozen and dried for 2 h.

#### 2.2.2. Fabrication of Urea Biosensor

An H^+^ ion transducer was first developed by mixing adequate amounts of the *n*-BA monomer, HDDA, HI, DMPP, and NaTFPB. The mixture (4 μL) was then drop-coated onto the Ag|AgCl SPE and photo-cured for 3 min under constant nitrogen gas flow. Immobilization of the urease enzyme onto C_60_-COOH was performed by adding 2 mL of a mixture of 5 mg/mL urease solution and 0.1 M EDC to 2.0 mg of modified fullerenes. The urease solution contained 5.0 mg urease in 1.0 mL PBS (pH 7.0, 10^−3^ M). The mixture was then kept in the fridge for 24 h. The obtained C_60_-urease was centrifuged, washed with deionized water, and separated from the solution. Then, 1.0 mg of C_60_-urease was dispersed in 50 μL of deionized water and deposited onto the H^+^ ion sensor.

#### 2.2.3. Characterization of Fullerene-Immobilized Urease

The shape and size of C_60_-urease was investigated by FESEM-EDX at an acceleration voltage of 3.00 kV. Dry C_60_-urease was placed on a stick. The size and distribution of urease on the surface of fullerene were determined based on random selection using the SE2 detector. The percentages of nitrogen, oxygen, and carbon were measured by EDX. The characteristics of nitrogen show that urea was immobilized on the fullerene surface.

The functional groups of urease explored by FTIR. The modified functionalized fullerene mixed with dried KBr and FTIR instrument detected prominent peaks for urease and carboxylic groups.

#### 2.2.4. Leaching Test

The Bradford protein assay was conducted to ascertain the amount of immobilized urease that had leaked from the surface of fullerene. Standard protein solutions were prepared by mixing the BSA–protein standard solution with Bradford reagent. For this purpose, this method was utilized to get a standard calibration curve from 3 mL of Bradford reagent mix with five concentrations of BSA, from 0 mg/mL to 1.4 mg/mL. Furthermore, 3 mL of Bradford reagent was added to a sample with an unknown amount of urease on fullerene. The standard and unknown sample solutions were gently mixed by vortex device, which was followed by incubation at room temperature for approximately 45 min. The standard solutions and the unknown amount of urease was determined from the absorbance using a spectrophotometer at λ = 595 nm.

#### 2.2.5. Optimization of H^+^ Ion Transducer Response

Before the fabrication of the potentiometric urea biosensor, the H^+^ ion sensor/transducer was prepared and tested. Hydrogen ionophore was entrapped inside the *n*-BA matrix by photo-curing. The developed H^+^ ion transducer was tested against 10^−3^ M PBS in the pH range of 5.5 to 8.0 using a Ag|AgCl reference electrode with a gel bridge. The H^+^ ion sensor response was then plotted against the H^+^ ion concentration.

#### 2.2.6. Effect of pH and Buffer Concentration on Biosensor Response

The urease-nanoparticle-modified SPE were used with different buffer concentrations from 0.0001 M to 0.05 M in various pH values for potentiometric measurement. One prepared SPE electrode was dipped in various concentrations the urea solution with the same pH but in different buffer concentrations. The optimal buffer concentration was selected for use in the pH range of 6 to 8.

#### 2.2.7. Carbodiimide Reaction for Urease Immobilization

Immobilization of urease on C_60_-COOH was conducted using two types of carbodiimide-leaving group reagents, that is, EDC and DCC. EDC is a water-soluble carbodiimide wherein the carbodiimide–amine reaction was conducted in the aqueous reaction. Immobilization of urease using DCC required an alcoholic reaction medium. The functionalized fullerene immersed in 0.1 M EDC or DCC solution include urease enzyme for overnight to replace OH^−^ of carboxylic functional group.

### 2.3. Evaluation of Biosensor Response

#### 2.3.1. Dynamic Range, Sensitivity, and Detection Limit

The dynamic range, detection limits, and sensitivity parameters were determined using a urea solution at different concentrations, from 10^−6^ M to 10^−1^ M, in 5 × 10^−4^ M, PBS buffer (pH 7.0). The prepared SPE working electrodes were dipped in different concentrations of urea in 0.5 mM of PBS at pH 7. The response of the biosensor based on the potential in mV was plotted against the logarithmic concentration of urea. The modified SPE with the HI but without the immobilized urease was similarly tested.

#### 2.3.2. Response Time and Stability

The response time of the urease biosensor was investigated using different concentrations of the urea solutions, from 10^−6^ M to 10^−1^ M, in 5 × 10^−1^ mM PBS buffer pH 7.0. The diluted urea solution to be used as controls was prepared in PBS buffer at pH 7.0. The urease-modified SPE was dipped in a 10^−5^ M urea solution. The potential of the modified SPE was recorded against the reference electrode. The calculated volume of 1 M urea was added to the diluted urea solution. The response time was recorded when the signal was stable, with less than 1 mV for three continuous measurements. This process was continuously performed until 10^−1^ M of urea solution was obtained. The modified SPE biosensors with the urease enzyme were investigated in different solutions of urea at different incubation times to obtain less than 5% decline in the sensitivity of SPE.

#### 2.3.3. Interference Study

Various methods can be used to determine the amount and type of interferences. Some cations such as Na^+^, K^+^, Ca^2+^, Mg ^2+^, and NH_4_^+^ were investigated via the separation solution method (SSM). The responses of the urease-modified SPEs in different concentrations of urea were compared with the same SPE in different concentrations of interference cation solutions within the same range. The response of modified SPEs in all interference solutions was plotted against a variety of concentrations.

#### 2.3.4. Repeatability and Reproducibility

Five different urea biosensors were used in different concentrations of urea solutions. Each fabricated urea biosensor was utilized four times to determine the amount of urea in various urea solutions of different concentrations. The relative standard deviation of repeatability and reproducibility was obtained based on the statistical rules and after calculating the sensitivity of each electrode.

#### 2.3.5. Real Sample Analysis

Urine was used to investigate the result of the fabricated electrode, after which the biosensor was compared using the standard method. UV-visible (Vis) spectroscopy is a standard method for measuring the amount of urea in urine. The absorbance of a variety of standard urea solutions from 0.04 mg urea/mL to 0.4 mg urea/mL was determined by UV-Vis spectrometry at 420 nm. The amount of urea in urine was calculated using the obtained calibration curve. The diluted urine solution was measured by three different fabricated electrodes to compare results from the standard and potentiometric methods.

## 3. Results and Discussion

### 3.1. Characterization of Modified Fullerene

**Figure 1.**
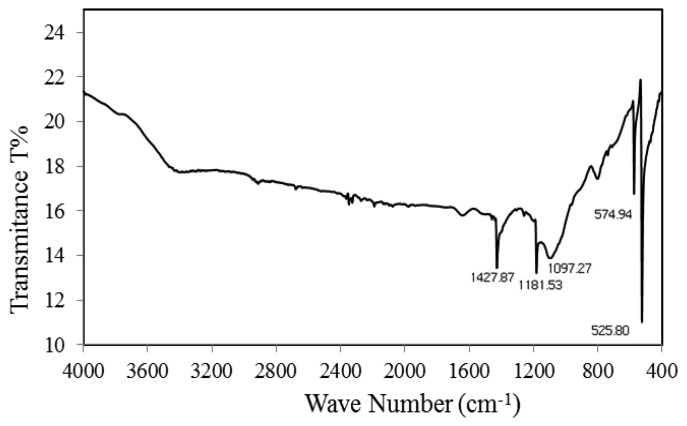
FTIR spectra of pristine fullerene.

Prominent fullerene peaks were observed at 525, 574, 1,097, 1,181, and 1,427 cm^−1^ by FTIR spectroscopy (Figure 1). The fullerene C60 has the highest symmetry of any known molecule. The point group symmetry of fullerene is I_h_. Although there are 174 vibrational degrees of freedom (3N-6) for each C60 molecule, the icosahedral symmetry of the fullerene C60 gives rise to a number of degenerate modes, so that only 46 frequency modes are expected for this molecule. Of these, four are infrared-active and 10 are Raman-active, whereas the remaining modes are optically inactive. Thus, the infrared spectrum of C60 is very simple, consisting of four modes with F_lu_ symmetry observed at frequencies of 527 [F_1u_ (1)], 576 [F_1u_ (2)], 1182 [F_1u_ (3)] and 1429 [F_1u_(4)] cm^−m^.

The 527 and 576 cm^−1^ modes are associated with a primarily radial motion of the carbon atoms, while the 1,182 and 1,427 cm^−1^ modes are essentially associated with a tangential motion of the carbon atoms. The most characteristic vibrational mode is the pentagonal ‘pinch’ mode at 1,427 cm^−1^. The temperature can affect a little shift in the position of the peaks. The well expressed absorption peaks indicate the four IR-active F_1u_ modes at 520, 572, 1,188, and 1,427 cm^−1^ in very good agreement with quantum chemical calculations on the vibrational spectrum reported [25–28].

**Figure 2.**
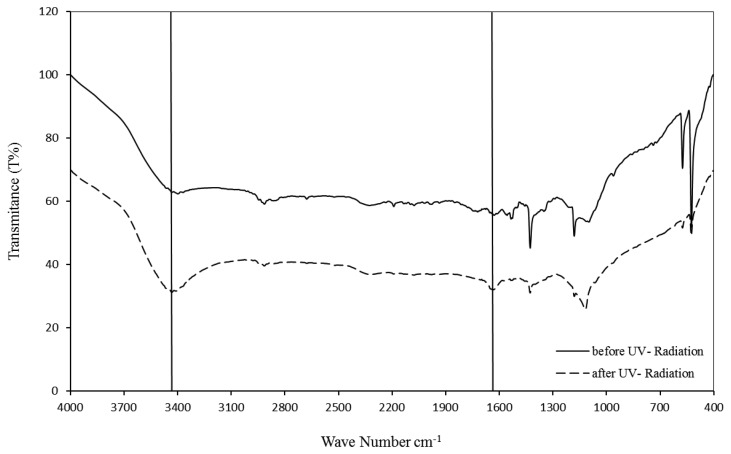
Peaks of −OH and C=O groups are clearer in C_60_-COOH after sonication and UV radiation in strong acids.

Sonication and UV radiation treatments of unmodified fullerene with strong acids at 75 °C produced a carboxyl functional group on the fullerene nanomaterial. The prominent peaks of unmodified fullerene were smaller or completely absent, whereas the peaks of –OH and C=O functional groups were more visible at 3,450 and 1,650 cm^−1^. The amount of C_60_-COOH used was the same in the preparation of samples via dried potassium bromide KBr for FTIR before and after UV irradiation. With the same amount of unmodified fullerene at the start and the same conditions in the process, after UV irradiation the prominent peak of fullerene is reduced and the OH peak is more obvious than before UV irradiation. UV light acts as an oxidation accelerator [29] (Figure 2).

**Figure 3.**
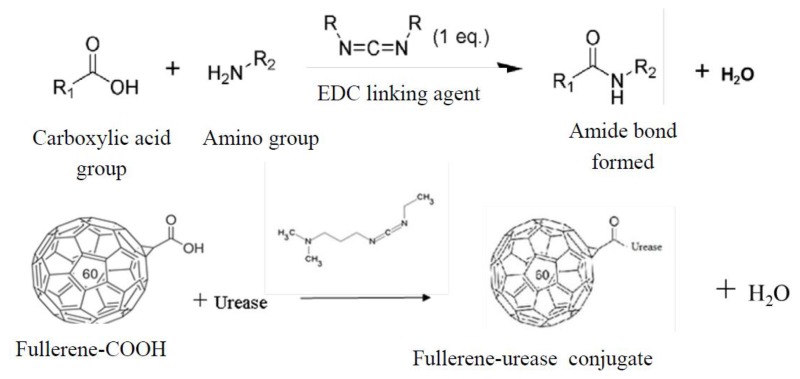
Schematic mechanism of reaction between modified fullerene and urease enzyme.

**Figure 4.**
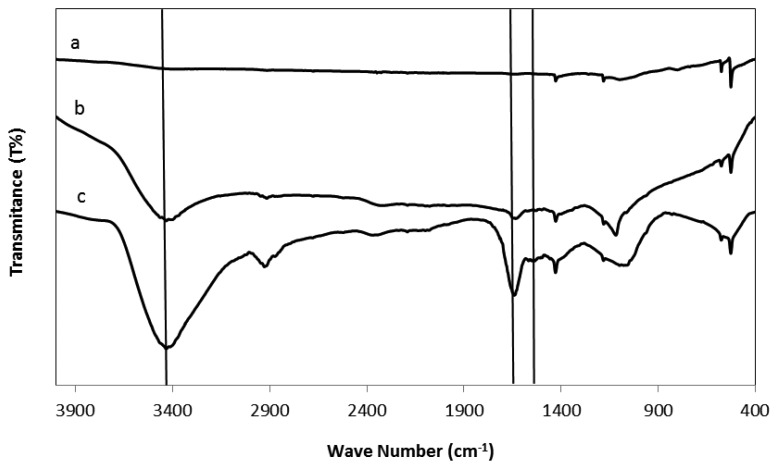
FTIR spectra of unmodified fullerene (**a**), C_60_-COOH (**b**), and C_60_-urease (**c**).

In the case of urease immobilization on modified fullerene, no direct chemical interaction occurs between the –COOH group of C_60_-COOH and the –NH_2_ of the urease enzyme. EDC and DCC are two potential carbodiimide-leaving groups that could connect –NH_2_ to –COOH via an amide covalent bond (–CONH–). EDC is typically employed in the pH range of 4.0 to 6.0. The linking agents EDC and DCC bind to carbonyl functional group of carboxylic group from modified fullerene and OH ^−^ leaves the molecule. Afterward, amino group of urease forms bonds with the carboxylic group of modified fullerene. The mechanism reaction is shown in Figure 3. This urease immobilization process on the fullerene molecules can be seen in the FTIR spectra in Figure 4.

Based on calibration from BSA protein assay using Bradford reagent, only a small amount of 0.1% of urease was leached from C_60_-urease conjugate, which indicates that urease had effectively attached to the fullerene surface.

EDX spectra normally display peaks corresponding to the energy levels for which the most X-rays have been received. Each of these peaks is unique to an atom, and therefore corresponds to a single element. The higher a peak in a spectrum, the more concentrated the element is in the specimen. The atomic percentages of carbon, oxygen, and nitrogen are 78.10%, 8.66%, and 13.24%, respectively. The EDX nitrogen peak at 0.4 kV can proved that urease has been attached on surface.

### 3.2. Optimization of H^+^ Ion Transducer Response

Changes in the pH that were caused by the enzymatic action of C_60_-urease were measured by the hydrogen ionophore-containing polyacrylate membrane. This membrane was deposited on the Ag|AgCl SPE electrode and functioned as an H^+^ ion transducer. The response of the H+ ion transducer was evaluated in 5 × 10^−4^ M PBS with different pH values before the immobilization of C_60_-urease on the electrode was carried out. The potential difference (EMF, in mV) of the H^+^ ion sensor was proportional to the pH values. The sensitivity of the H^+^ ion transducer was 60.18 ± 0.89 mV/decade (*R*^2^ = 0.9998), which was close to the Nernstian value. Thus, the results indicating that the developed H^+^ ion transducer was reliable for the further development of potentiometric urea biosensor.

### 3.3. Effect of Carbodiimide Reagents

**Figure 5.**
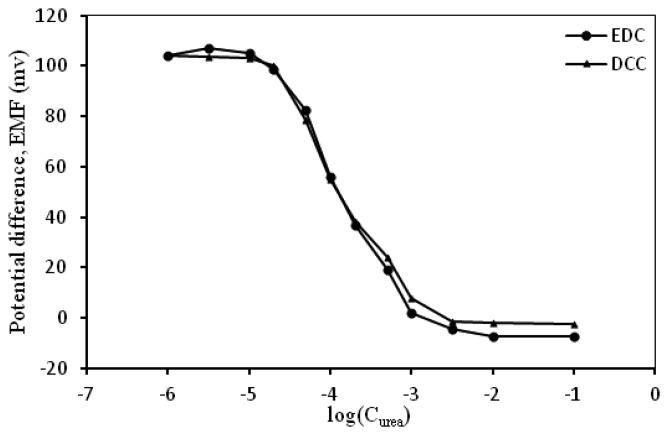
Difference in sensitivity when C_60_-COOH was modified by using DCC or EDC.

When EDC was employed for the fabrication of urea biosensor, the sensitivity of the biosensor was close to the Nernstian value (Table 1). As the activity of urease decreased in the alcoholic medium, lower sensitivity of urea biosensor was obtained using water-insoluble DCC (Figure 5). Incubation of urease was performed after removing and washing DCC from a mixture of C_60_-COOH. EDC and urease enzyme were simultaneously added to C_60_-COOH.

### 3.4. Effect of pH and Buffer Concentration on Biosensor Response

The enzymatic reaction of urease is based on the following reaction:

$${\left({\text{NH}}_{2}\right)}_{2}\text{CO}+3{\text{H}}_{2}\text{O}\;\underset{\to }{\text{Urease}}{2{\text{NH}}^{+}}_{4}+{\text{OH}}^{-}+{{\text{HCO}}^{-}}_{3}$$

**Figure 6.**
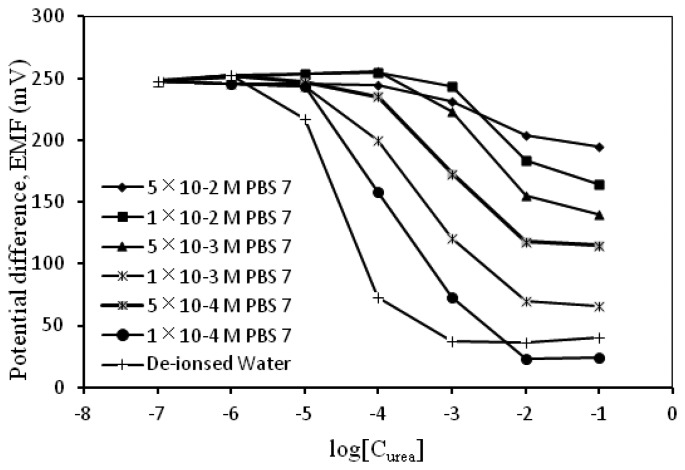
Effect of buffer concentration on the response of the urea biosensor.

The immobilized urease catalyzed the hydrolysis of urea in the sample and produced OH^−^ ions, which altered the pH of the sample solution. The study on the effect of buffer concentrations was performed by varying the concentrations of PBS (pH 7) from 0.0001 M to 0.05 M. At a very low concentration of urea, the OH^−^ ion reacted with buffer; thus, the concentration of the buffer became important, under the condition the buffer capacity could not maintain the pH and the sensitivity increased, e.g., the sensitivity values increased to 74–79 mV/decade (Table 2). At high concentrations of the buffer, the slope of sensitivity decreased because of the OH^−^ ion reacted with buffer. As shown in Table 2 and Figure 6, sensitivity was sufficient at high concentrations of the buffer; however, at low concentrations, the slope is greater than the Nernstian value. Based on the results in Table 2, the suitable concentrations for urea detection were 0.001 and 0.0005 M PBS because their observed sensitivities were close to the Nernstian value, *i.e.*, 58–60 mV/decade (Table 2).

Different concentrations of urea were measured at a series of pH values using 5 × 10^−4^ M PBS. The response of the biosensor as the pH medium varied from pH 2 to 10. The optimum pH range of the biosensor was obtained between pH 6.0 and 8.0 that is shown in Table 3. The sensitivities of the biosensor were relatively high, ranging from pH 6.0 to 7.0. The optimal dynamic range was seen at pH 7.5, but pH 7.0 was still chosen for the subsequent determinations.

### 3.5. Evaluation of Biosensor Response

**Figure 7.**
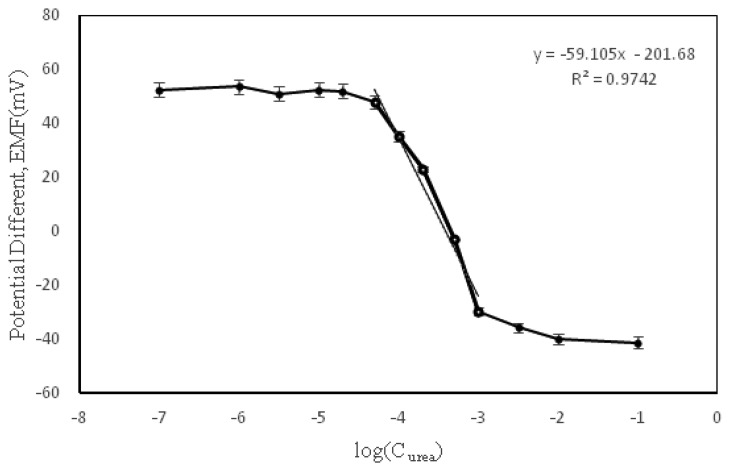
Variation in potential *versus* concentration of urea in 5 × 10^−4^ M PBS (pH 7.0).

By changing the concentration of urea, the amount of generated potential will also be changed. By measuring the amount of H^+^ using hydrogen ionophore in the first layer of SPE, the sensitivity or slope of the potential *versus* concentration of urea per decade was expected to be 59.67 ± 0.91 mV/decade based on the Nernstian equation. The variation potential in different concentrations of urea solution is shown in Figure 7. Based on the slope of dynamic range and variance, the electrode had good sensitivity to the amount of urea solution. The dynamic range was between 2.31 × 10^−3^ M to 8.28 × 10^−5^ M. A urea biosensor based on a piezoelectric quartz crystal microbalance (QCM) and coated C60-cryptand urease reported has the linear response range to urea from 10^−1^ to 10^−4^ M [21]. Thus, the biosensor in this work demonstrated a lower detection limit when compared with QCM urea biosensor.

In enzyme sensors, the change in electrode potential with time is directly proportional to the reaction rate. Brady and Carr [30] showed that potentiometric electrodes display a linear response to bulk substrate concentration only up to about one-tenth of the value of Michaelis constant (*K*_M_). This linear range is essentially independent of the amount of immobilized enzyme. pH is one of the important factors in the maximum activity of the enzyme. The optimum pH may not be the same as the soluble enzyme because it depends on the carrier used.

**Figure 8.**
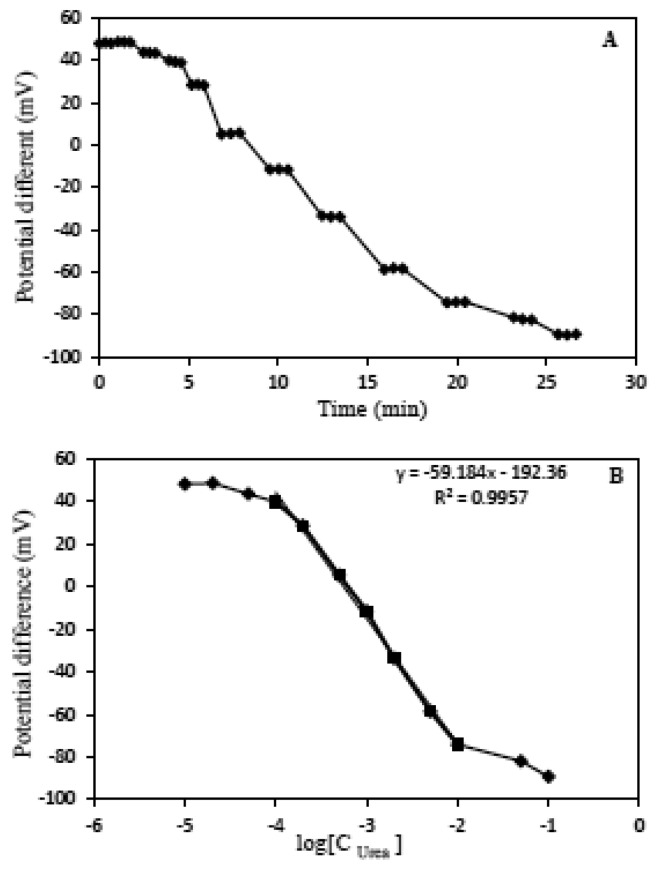
Response time of the urea potentiometric biosensor when the concentrations of urea were changed step by step from 10^−5^ M–10^−1^ M (**A**); The observed potential in (A) was plotted against urea concentrations from 10^−5^ M–10^−1^ M (**B**).

In Figure 8, A illustrates that the response time is less than 2 min for each determination and the responses are constant within the dynamic range area and B shows the Nernstian response to various amounts of urea concentrations.

### 3.6. Shelf Life of the C_60_-Urease Electrode

Three samples were measured in the different urea solutions of concentrations at consecutive incubation times. The sensitivity decreased with the passage of time. The response of the urea biosensor was decreased by only 5% after 140 days. The storage conditions serve an effective function in response reduction. The modified SPE were kept in 4 °C in a refrigerator under dry condition. The activity of the enzyme decreased with time, thus reducing sensitivity. The rate of recline was 2.18 × 10^−2^ ΔmV/decade per day.

### 3.7. Interference Study

**Figure 9.**
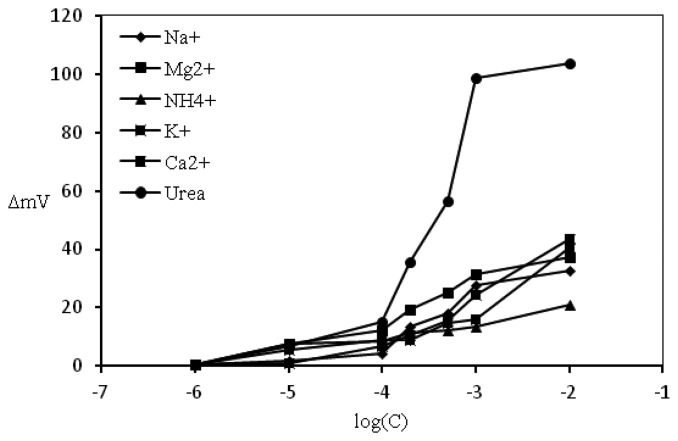
Comparison of sensitivity for the urea biosensor electrode to urea and some other cations.

As shown in Figure 9, minimal interference was caused by the ions and urea could be measured when other cations are absent. Common cations were chosen to investigate their effect on the response to urea. One SPE was used for all ion solutions in various concentrations. The plot of variation in potential based on change in concentration of the urea solution (Figure 9) shows that only minor response was observed when the cations were used and this response may be induced by the pH ionophore and the lipophilic salt that contained in the pH transducer membrane.

To investigate the repeatability and reproducibility of the five fabricated urea enzyme sensors, they were measured at 0.5 mM of PBS 7.0 at a variety of concentrations of urea solutions. The repetability and reproducibility (*n* = 5 and 4, respectively) measured for each sample. The results show that the sensors have good repeatabilty and reproducibility with 1.36 and 1.53 RSD% respectively.

### 3.8. Real Sample

The three fabricated urea biosensors were used for 20 times the diluted urine solution. The results are compared in Table 4. The differences between the results from UV-Vis standard method and potentiometric bisoensor method is less than 5%. This shows that the biosensor developed here can be used to determine urea with confidence.

## 4. Conclusions

Fullerene has been successfully modified for use as a matrix for the immobilization of urease for the development of a new potentiometric urea biosensor. The immobilization of urease on fullerene was also characterized. The developed potentiometric enzyme electrode showed linearity to urea in concentrations of 1.2 mM to 0.042 mM in PBS at pH 7.0. The successful biosensor performance is attributed to the immobilization of the fullerene-urease bio-conjugate on an acrylic based hydrogen ion sensitive membrane. The use of fullerene-urease bio-conjugate and the good adhesion acrylic membrane has prevented the leaching of urease enzyme and sustained the activity of the urease and this is reflected in the stability of the urea biosensor for up to 140 days.

## Figures and Tables

**Table 1.** Effect of DCC or EDC on the response of the C_60_-urease-modified SPE electrode.

**Type of Carbodiimide Leaving** **Group Reagent**	**Average Sensitivity** **of Samples**	**Dynamic Linear Range** **(M)**
DCC	54.6 ± 0.17	10^−3^–10^−5^(*R*^2^ = 0.9854)
EDC	58.8 ± 0.75	10^−3^–10^−5^(*R*^2^ = 0.9846)

**Table 2.** Effect of PBS (pH 7) concentration on the performance of urea biosensor sensitivity.

**Concentration of PBS (M)**	**0.05**	**0.01**	**0.005**	**0.001**	**0.0005**	**0.0001**	**Deionized Water**
Sensitivity (mV/Decade)	17.6	39.4	50.6	60.1	58.8	74.6	79.1
Linear range (M)	10^−4^–10^−1^	10^−3^–10^−1^	10^−4^–10^−2^	10^−5^–10^−2^	10^−5^–10^−2^	10^−5^–10^−2^	10^−6^–10^−3^
R^2^	0.963	0.917	0.959	0.988	0.998	0.986	0.929

**Table 3.** Influence of pH on biosensor response using 5 × 10^−4^ M PBS.

**pH**	**6.0**	**6.5**	**7.0**	**7.5**	**8.0**
Sensitivity	56.0	59.3	60.8	53.5	38.8
Dynamic range (M)	3.7 × 10^−3^–8.9 × 10^−6^	1.3 × 10^−2^–2.5 × 10^−4^	1.2 × 10^−3^–4.2 × 10^−5^	4.16 × 10^−2^–2.0 × 10^−4^	8.9 × 10^−3^–7.7 × 10^−5^
R^2^	0.9782	0.9816	0.9745	0.9899	0.9911

**Table 4.** Comparison of the responses of fabricated electrode by potentiometric method and UV-Vis standard method.

	**Real Amount (M) Std.** **Method**	**Real Amount (M) Potentiometric** **Method**	**Differences (%)**
Full-U/SPE 1	0.018 ± 0.003	0.017 ± 0.005	−3.61
Full-U/SPE 2	0.018 ± 0.003	0.019 ± 0.006	4.91
Full-U/SPE 3	0.018 ± 0.003	0.017 ± 0.004	−4.58
